# Prevalence of depressive symptoms and correlates among individuals who self-reported SARS-CoV-2 infection after optimizing the COVID-19 response in China

**DOI:** 10.3389/fpubh.2023.1268799

**Published:** 2024-01-08

**Authors:** Liangjia Wei, Jiao Qin, Zhifeng Lin, Xinju Huang, Jinfeng He, Dee Yu, Fei Zhang, Sisi Li, Ping Cen, Mu Li, Tong Luo, Rongjing Zhang, Shanmei Zhong, Cai Qin, Zeyu Li, Yuan Yang, Huiqi Pan, Mengdi Zhao, Xiaoqiong Wu, Junjun Jiang, Hao Liang, Li Ye, Bingyu Liang

**Affiliations:** ^1^Guangxi Key Laboratory of AIDS Prevention and Treatment, School of Public Health, Guangxi Medical University, Nanning, Guangxi, China; ^2^International School of Public Health and One Health, Hainan Medical University, Haikou, Hainan, China; ^3^Nanning Center for Disease Control and Prevention, Nanning, Guangxi, China; ^4^Collaborative Innovation Centre of Regenerative Medicine and Medical BioResource Development and Application Co-constructed by the Province and Ministry, Life Science Institute, Guangxi Medical University, Nanning, Guangxi, China

**Keywords:** COVID-19, depressive symptoms, healthcare services, optimizing the COVID-19 response, PHQ-9 scale

## Abstract

**Background:**

The burden of depression symptoms has increased among individuals infected with SARS-CoV-2 during COVID-19 pandemic. However, the prevalence and associated factors of depressive symptoms among individuals infected with SARS-CoV-2 remain uncertain after optimizing the COVID-19 response in China.

**Methods:**

An online cross-sectional survey was conducted among the public from January 6 to 30, 2023, using a convenience sampling method. Sociodemographic and COVID-19 pandemic-related factors were collected. The depression symptoms were assessed using the Patient Health Questionnaire-9 (PHQ-9). Logistic regression analysis was performed to explore the associated factors with depressive symptoms.

**Results:**

A total of 2,726 participants completed the survey. The prevalence of depression symptoms was 35.3%. About 58% of the participants reported experiencing insufficient drug supply. More than 40% of participants reported that they had missed healthcare appointments or delayed treatment. One-third of participants responded experiencing a shortage of healthcare staff and a long waiting time during medical treatment. Logistic regression analysis revealed several factors that were associated with depression symptoms, including sleep difficulties (OR, 2.84; 95% CI, 2.34–3.44), chronic diseases (OR, 2.15; 95% CI, 1.64–2.82), inpatient treatment for COVID-19 (OR, 3.24; 95% CI, 2.19–4.77), with COVID-19 symptoms more than 13 days (OR, 1.30, 95% CI 1.04–1.63), re-infection with SARS-CoV-2 (OR, 1.52; 95% CI, 1.07–2.15), and the increased in demand for healthcare services (OR, 1.32; 95% CI, 1.08–1.61).

**Conclusion:**

This study reveals a moderate prevalence of depression symptoms among individuals infected with SARS-CoV-2. The findings underscore the importance of continued focus on depressive symptoms among vulnerable individuals, including those with sleeping difficulties, chronic diseases, and inpatient treatment for COVID-19. It is necessary to provide mental health services and psychological interventions for these vulnerable groups during the COVID-19 epidemic.

## Introduction

Accumulating evidence showed that a high prevalence of mental health issues due to the COVID-19 pandemic (1–5). Depressive symptoms are prevalent in the public during the COVID-19 pandemic. Previous studies indicated that the COVID-19 pandemic has caused high levels of depressive symptoms, with a pooled prevalence ranging from 23% to 43% (6–8). It has been reported that COVID-19-related challenges disproportionately impact the experience of depressive symptoms among various populations. Previous studies suggested that healthcare workers (9), individuals with chronic diseases (10), students (11), and pregnant women (12) were more susceptible to depression symptoms. Furthermore, several risk factors, such as sociodemographic characteristics (e.g., younger age, female gender, lower income) and pandemic-related factors (e.g., COVID-19 exposure factors, shortage of resources, less social contact), were associated with depressive symptoms during the pandemic (13–19).

However, it is crucial to note that these findings display heterogeneity, attributable to differences in target populations, sampling methods, disease prevalence in local, policy stringency, and cultural context.

Notably, there is an elevated risk of developing incident depression symptoms among the SARS-CoV-2 infected people (13, 20). For instance, a study conducted in South Sinai, Egypt, revealed that the prevalence of depression symptoms among SARS-CoV-2 patients was 46.3% (21). This research found that patients who experienced hospitalization were more likely to exhibit symptoms of depression (21). Furthermore, a systematic review and meta-analysis revealed that individuals infected with SARS-CoV-2 were more susceptible to developing depressive symptoms, with a pooled prevalence of 41.7%, while the general population reported a prevalence of 31.5% during the COVID-19 outbreak (22). This underscores the importance of paying attention to the mental health issues among the infected population during the pandemic. Several factors, including social isolation, psychological stress, chronic illness, and the severity of COVID-19, are acknowledged as potential contributory risk factors for developing depressive symptoms after SARS-CoV-2 infection (23).

On December 7, 2022, China implemented 10 new optimization measures in response to COVID-19. These measures notably reduced mobility restrictions on the population and adjusted the isolation methods for infected individuals, particularly those with asymptomatic or mild cases. Studies have shown that 80% to 90% of the public in China was infected with SARS-CoV-2 between December 2022 to January 2023 (24, 25). Certain factors may contribute to an increased risk of depression symptoms among individuals infected with SARS-CoV-2 after optimizing the COVID-19 response in China. For instance, people intentionally keep a social distance for fear of infecting SARS-CoV-2, leading to feelings of isolation and anxiety. Moreover, temporary job losses, alterations in working hours, and decreased salaries may cause a high prevalence of depression symptoms during the COVID-19 epidemic. As the epidemic continues, access to healthcare has become increasingly challenging, exacerbating anxiety and stress among the population. Taken together, these factors may be associated with an increased risk of depressive symptoms among infected individuals (13, 20, 26).

However, it is worth noting that there is limited reported evidence on the prevalence of depression symptoms and its associated factors among infected individuals after the optimization of the COVID-19 response in China. Therefore, we used an online survey to investigate the prevalence of depression symptoms and correlates among individuals who self-reported being infected with SARS-CoV-2. Furthermore, we also examined the access to healthcare services after the optimization of the COVID-19 response in China. This study aims to identify potential factors associated with depression symptoms after optimization the COVID-19 response in China. It may provide valuable insights for healthcare professionals to implement interventions aimed at improving mental health among individuals infected with SARS-CoV-2.

## Methods

### Study design and participants

A cross-sectional survey was conducted among the public in China from January 6 to 30, 2023, using a convenience sampling method.

The inclusion criteria for participants were (1) age ≥18 years and (2) the absence of a diagnosed mental disorder. The exclusion criteria were (1) age under 18 years and (2) a pre-existing mental disorder.

### Sample size and technique

Several studies have estimated the prevalence of depressive symptoms among individuals infected with SARS-CoV-2, using a score of PHQ-9 ≥10. The prevalence of depressive symptoms ranges from 31.6% to 52%, using a score of PHQ-9≥10 (27–29). Therefore, we chose a prevalence of 30% for calculating the sample size. A sample size of *n* = 2,065 was calculated by using PASS software version 15. Taking into account a rate of loss to follow-up of 10%, therefore, the sample size was at least 2,272.

**Figure 1:**
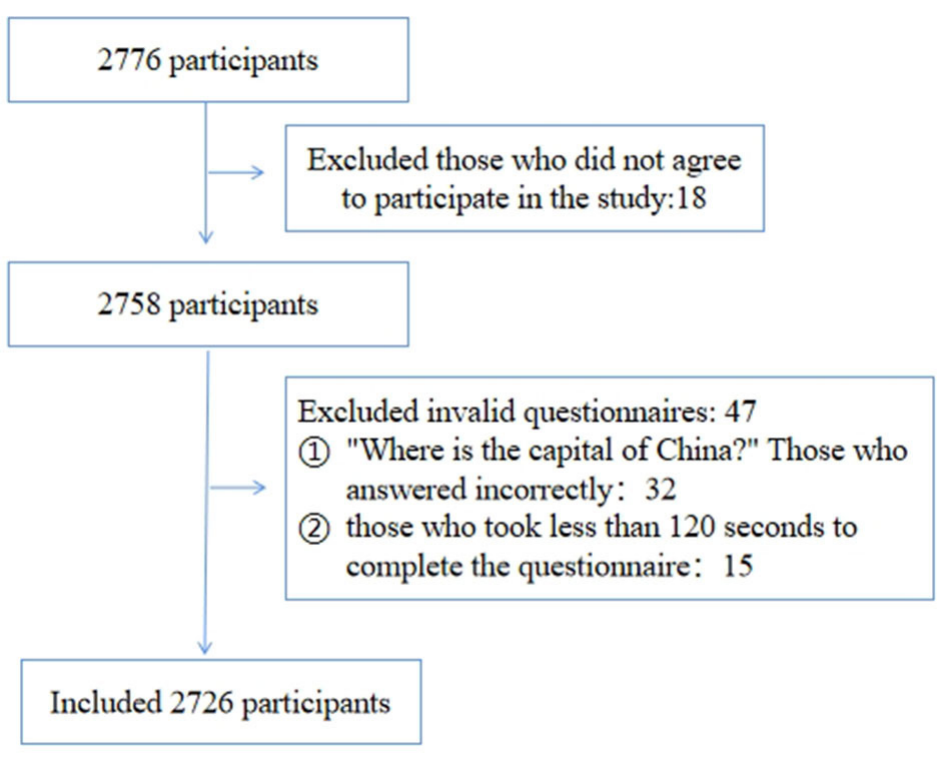
Study subjects flowchart.

A total of 2,791 completed the survey. Eighteen people were excluded from the survey as they refused to complete the questionnaire. Additionally, 47 participants were excluded due to their invalid responses. The final sample size included in the analysis was 2,726 (Figure 1).

### Procedure

The study used a convenience sampling method to enroll participants who were both available and willing to participate. Importantly, we hypothesized that depression symptoms among participants would decrease gradually over time after the initial implementation of the relaxed policy. Consequently, data was collected during the early period of optimizing the COVID-19 response in China, which coincided with the peak of SARS-CoV-2 infections characterized by a significant surge in the number of cases. We employed the “Questionnaire Star,” a platform based on WeChat, to administer the questionnaires (30). Participants were invited to complete the questionnaire by scanning the QR code. Moreover, to ensure the quality of data, quality control questions were included in the questionnaire.

The study was approved by the Medical Ethics Committee of Guangxi Medical University (20220206). All participants were provided with online informed consent. The survey was anonymous and did not collect any personally identifiable information. Nevertheless, participants were informed that they had the right to withdraw at any point during the survey.

### Measures

A structured questionnaire was composed of three sections: sociodemographic information, pandemic-related variables, and an assessment of depressive states.

### Sociodemographic variables

Self-reported sociodemographic data were collected for gender, age, ethnicity, education, monthly income, marital status, occupation, current residence area, residency status, home-to-healthcare facility commute time, and the history of chronic disease/smoking/drinking alcohol.

### Pandemic-related variables

**Figure 2:**
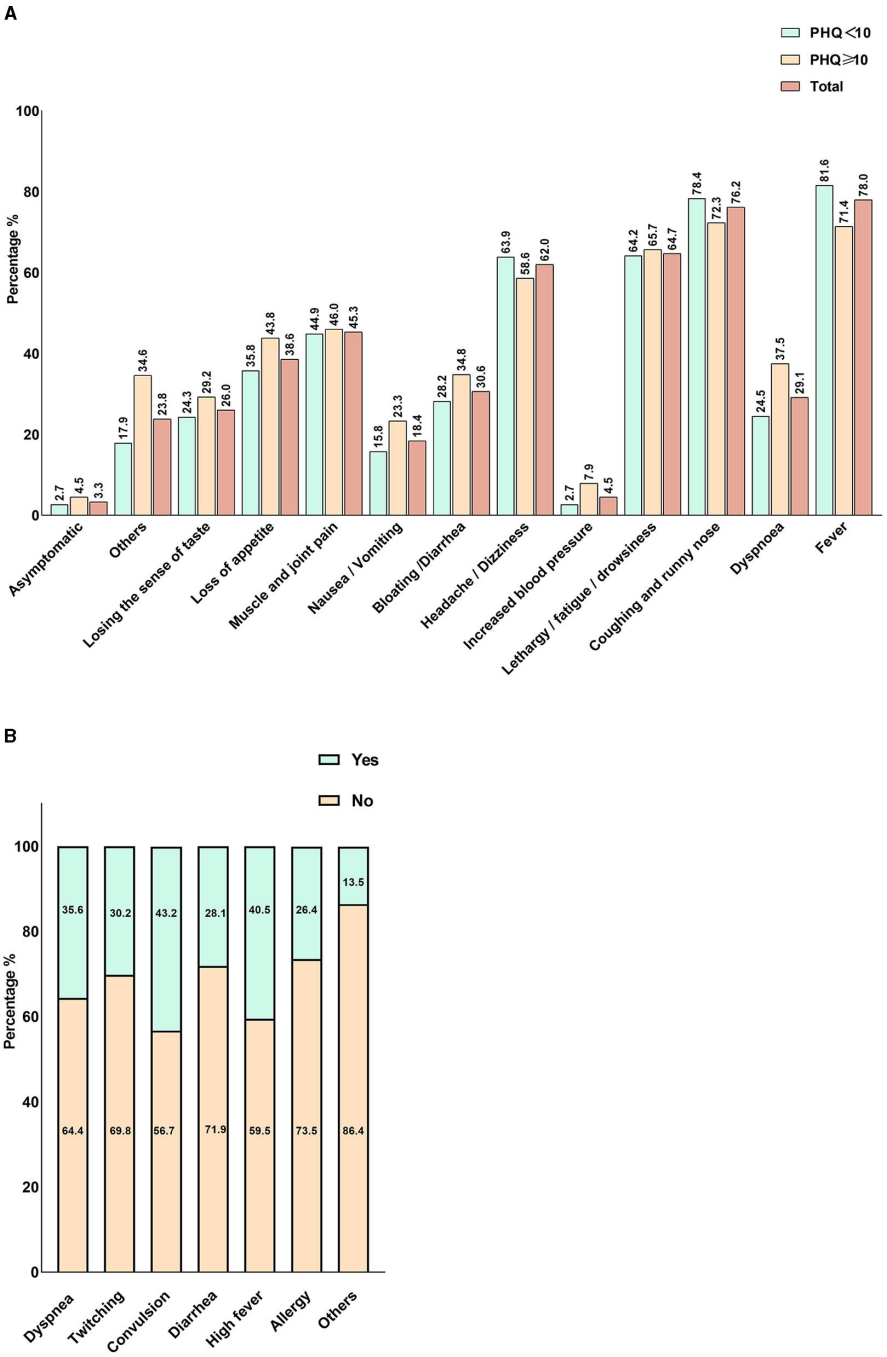
Self-reported COVID-19 Symptoms among participants and main symptoms for inpatient treatment. **(A)** The self-reported COVID-19 symptoms among participants. **(B)** The main symptoms for inpatient treatment. In the legend, “Yes” indicates the presence of the symptom, and “No” represents the absence of the symptom.

**Figure 3:**
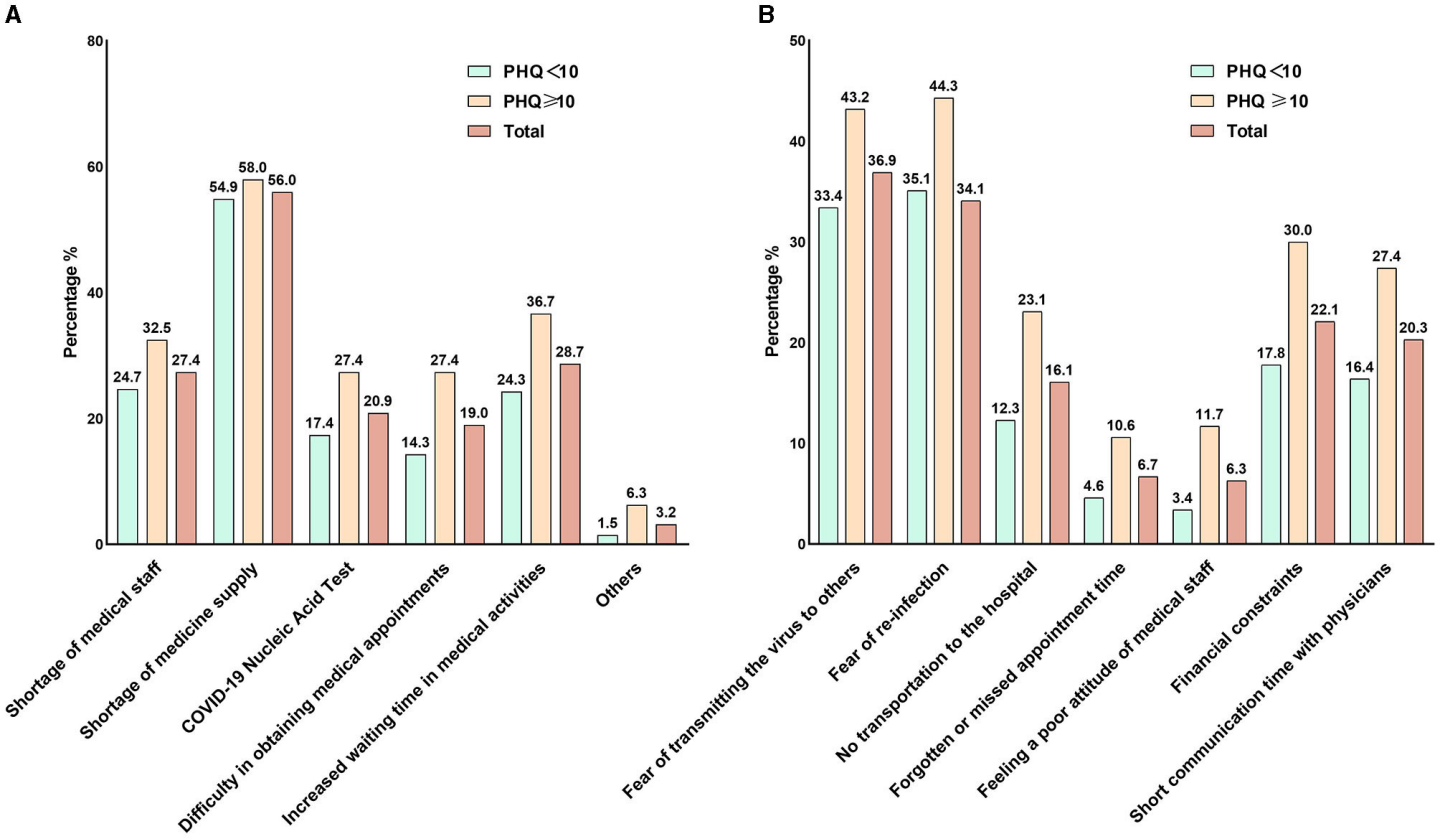
Barriers to accessing healthcare services and reasons for delayed or canceled medical appointments after optimizing the COVID-19 response in China. **(A)** Barriers to accessing healthcare services. **(B)** The reasons for delaying or canceling medical appointments.

This section addresses the behaviors or characteristics related to the pandemic after the optimization of the COVID-19 response in China and contains the following questions: the interruption of physical exercise (“Yes,” “No”); sleep difficulties (“Yes,” “No”); Change in utilization of healthcare services (“Same as before,” “More than before,” “less than before”); Change in healthcare costs (“the same as before,” “more than before,” “less than before”); the barriers in accessing health services are summarized in Figure 3A; Reasons for delayed or canceled medical attention are summarized in Figure 3B; COVID-19 vaccination status (“Completed zero dose,” “Completed one dose,” “Completed two doses,” “Completed three doses,” “Completed four doses”); High consumption of COVID-19-related news (“Yes,” “No”); perception of COVID-19 (“Very serious infectious disease,” “Common infectious disease,” “I don't know”); Re-infected with SARS-CoV-2 (“Yes,” “No”); Inpatient treatment for COVID-19 (“Yes,” “No”); length of COVID-19 symptom (“1 to 6 days,” “7 to 12 days,” “more than 13 days”); the self-reported COVID-19 symptoms are summarized in Figure 1. The reasons for inpatient treatment for COVID-19 are summarized in Figure 2.

### Measurement of depressive symptoms

The Patient Health Questionnaire-9 (PHQ-9) was used to estimate the prevalence of depression symptoms. It has been reported that the PHQ-9 scale has good reliability with Cronbach's alpha of 0.91 (31). A comprehensive meta-analysis demonstrated that a cut-off score of 10 for PHQ-9 maximized both sensitivity and specificity (32). The sensitivity and specificity of PHQ-9 were both 0.85 at thresholds of 10 (33). Moreover, PHQ-9≥10 is widely employed for estimating the prevalence of depression symptoms and has been published in high-impact journals (34, 35). Hence, the scores of PHQ-9 ≥10 were used to define the symptoms of depression in this study. Participants were divided into two groups: those with PHQ-9 scores ≥10 were classified as having depressive symptoms, while those with scores below 10 were considered as not having depressive symptoms.

The Chinese version and the original PHQ-9 scale are consistent, comprising nine items. It covers the following areas: (1) “No interest or pleasure in doing things,” (2) “feeling depressed, downhearted, or hopeless,” (3) “feeling tired or having no energy,” (4) “feeling bad/frustration/failure about myself, or the sense that you let your family down,” (5) “Thoughts of dying or hurting yourself in some way,” (6) “difficulty sleeping or sleeping too much,” (7) “poor appetite or overeating,” (8) “difficulty concentrating,” and (9) “slow movement/speech or irritability or fidgeting.” The total score is calculated by summing the scores for each question, which range from 0 to 27. The reliability of this study was 0.92 (36, 37).

### Statistical analysis

All data were imported into Microsoft Excel 2019 for collation. Data were statistically analyzed using SPSS version 25.0 (IBM, Armonk, NY, United States). Descriptive information including the sociodemographic and pandemic-related predictor variables were provided as percentages. Participants were categorized into two groups: those who reported SARS-CoV-2 infection were categorized as the SARS-CoV-2 infected group, while others were considered as the non-SARS-CoV-2 infected group. The chi-square test was employed to explore the differences of these two groups. For multivariate analysis, binary logistic regression was used to examine the associated factors with symptoms of depression among all participants.

Then, we compared differences between participants with and without depressive symptoms among individuals with the SARS-CoV-2 infection using the chi-square test. Variables with a *p* < 0.05 in analyses were included in multivariate analyses. Subsequently, for multivariate analysis, binary logistic regression was performed to explore the factors associated with depressive symptoms after assessing multicollinearity. Variables with variance inflation factors (VIF) >5 or tolerances <0.1 were excluded from the multivariate analysis. For multivariate analysis using the backward conditional method, *P* < 0.05 was used as the cutoff for entering the model. In addition, percentages were used to describe self-reported COVID-19 symptoms and symptoms for hospitalization. Barriers to healthcare access and reasons for delayed or canceled medical appointments were also described as percentages. Bar charts were employed for visualization.

## Results

Table 1 presents the Sociodemographic of participants. A total of 2,726 participants completed the survey, with 2,332 participants who self-reported being infected with SARS-CoV-2 (a positive SARS-CoV-2 test or clinical symptom-diagnosed) and 394 participants were not infected with SARS-CoV-2. The age ranges from 18 to 81 years (M = 29.5 years, SD = 11.1). More than half of the participants were female (62.5%) and single (60.6%). A total 63.4% of the participants belonged to the Han ethnicity, and 61.3% of the participants had a college or undergraduate education. The majority of participants (72.9%) reported a commute time of <30 min from their homes to healthcare facilities. Only 13.7% of the participants reported that they had a history of chronic disease. Additionally, the prevalence of depression symptoms among participants infected with SARS-CoV-2 was 33.3%. Additional details are provided in Table 1. Furthermore, participants who self-reported being infected with SARS-CoV-2 had a 2-fold higher risk of depression symptoms compared to non-infected individuals (Supplementary Table 1).

**Table 1:** Socio-demographic of the participants.

**Characteristic**	**Total (*n =* 2,726), No. (%)**	**Self-reported SARS-CoV-2 infection**		***P*-value**
		**Yes (*****n** =* **2,332)**	**No (*****n** =* **394)**	
**Age**				0.165
≤ 24	1,178 (43.2)	993 (42.6)	185 (47.0)	
25–34	843 (30.9)	736 (31.6)	107 (27.2)	
≥35	705 (25.9)	603 (25.9)	102 (25.9)	
**Gender**				<0.001
Male	1,021 (37.5)	842 (36.1)	179 (45.4)	
Female	1,705 (62.5)	1,490 (63.9)	215 (54.6)	
**Ethnicity**				0.505
Han	1,729 (63.4)	1,485 (63.7)	224 (61.9)	
Zhuang and other	997 (36.6)	847 (36.3)	150 (38.1)	
**Education**				0.019
High school or below	486 (17.8)	396 (17.0)	90 (22.8)	
College or undergraduate	1,672 (61.3)	1,445 (61.9)	227 (57.6)	
Postgraduate	568 (20.8)	491 (21.1)	77 (19.5)	
**Monthly income**				0.080
<2,000	1,164 (42.7)	976 (41.9)	188 (47.7)	
2,000–4,999	804 (29.5)	694 (29.8)	110 (27.9)	
≥5,000	758 (27.8)	662 (28.4)	96 (24.4)	
**Marital status**				0.033
Married	1,073 (39.4)	937 (40.2)	136 (34.5)	
Single (unmarried, Cohabitation/divorced/widowed)	1,653 (60.6)	1,395 (59.8)	258 (65.5)	
**Occupation**				0.032
Farmer or worker	230 (8.4)	196 (8.4)	32 (8.6)	
Company or government employee	828 (30.4)	716 (30.7)	112 (28.4)	
Business or self-employed	540 (19.8)	479 (20.5)	61 (15.5)	
Unemployed	75 (2.8)	59 (2.5)	16 (4.1)	
Student	1,053 (38.6)	882 (37.8)	171 (43.4)	
**Current residence area**				0.001
Urban	2,138 (78.4)	1,855 (79.5)	283 (71.8)	
Rural	588 (21.6)	477 (20.5)	111 (28.2)	
**Residential status**				0.203
Live alone	475 (17.4)	408 (17.5)	67 (17.0)	
Live with family	1,783 (65.4)	1,512 (64.8)	271 (68.8)	
Live with others	468 (17.2)	412 (17.7)	56 (14.2)	
**The home-to-healthcare facility commute time**				0.921
<30 min	1,987 (72.9)	1,699 (72.9)	288 (73.1)	
≥ 30 min	739 (27.1)	633 (27.1)	106 (26.9)	
**Chronic diseases**				0.158
No	2,353 (86.3)	2,004 (85.9)	349 (88.6)	
Yes	373 (13.7)	328 (14.1)	45 (11.4)	
**Smoking**				0.005
No	2,216 (81.3)	1,916 (82.2)	300 (76.1)	
Yes	510 (18.7)	416 (17.8)	94 (23.9)	
**Drinking**				0.145
No	2,025 (74.3)	1,744 (74.8)	281 (71.3)	
Yes	701 (25.7)	588 (25.2)	113 (28.7)	
**The interruption of physical exercise**				0.109
Yes	962 (35.3)	837 (35.9)	125 (31.7)	
No	17,664 (64.7)	1,495 (64.1)	269 (68.3)	
**Sleep difficulties**				<0.001
No	1,743 (63.9)	1,437 (61.6)	306 (77.7)	
Yes	983 (36.1)	895 (38.4)	88 (22.3)	
**Changes in medical expenses**				<0.001
The same as before	1,285 (47.1)	1,048 (44.9)	237 (60.2)	
More than before	1,332 (48.9)	1,197 (51.3)	135 (34.3)	
Less than before	109 (4.0)	87 (3.7)	22 (5.6)	
**Change in healthcare services**				<0.001
The same as before	1,483 (54.4)	1,221 (52.4)	262 (66.5)	
More than before	1,098 (40.3)	1,000 (42.9)	98 (24.9)	
Less than before	145 (5.3)	111 (4.8)	34 (8.6)	
**COVID-19 vaccination status**				<0.001
Completed 0 dose	40 (1.5)	33 (1.4)	7 (1.8)	
Completed 1 dose	25 (1.3)	33 (1.4)	2 (0.5)	
Completed 2 doses	350 (12.8)	298 (12.8)	52 (13.2)	
Completed 3 doses	2,051 (75.2)	1,783 (76.5)	268 (68.0)	
Completed 4 doses	250 (9.2)	185 (7.9)	65 (16.5)	
**High consumption of COVID-19-related news**				0.894
No	609 (22.3)	522 (22.4)	87 (22.1)	
Yes	2,117 (77.7)	1,810 (77.6)	307 (77.9)	
**Perception of COVID-19**				0.008
Very serious infectious disease	2,300 (84.4)	1,977 (84.8)	323 (82.0)	
Common infectious disease	337 (12.4)	289 (12.4)	48 (12.2)	
I don't know	89 (3.3)	66 (2.8)	23 (5.8)	
**Scores of PHQ-9**				
PHQ-9 <10	1,826 (66.9)	1,508 (64.7)	318 (80.7)	<0.001
PHQ-9≥10	900 (33.3)	824 (35.3)	76 (19.3)	

Table 2 shows the differences in the distribution of sociodemographic and epidemic-related factors between depressed and non-depressed individuals infected with SARS-CoV-2. There were significant differences in sociodemographic and COVID-19-related factors, such as age, education, occupation, current residence area, the home-to-healthcare facility commute time, history of chronic illness, smoking, drinking, sleep difficulties, change in healthcare services, change in healthcare costs, vaccines, attitudes toward COVID-19, re-infected with SARS-CoV-2, duration of COVID-19 symptoms, inpatient treatment for COVID-19.

**Table 2:** Associated factors with depression among participants with Self-reported SARS-CoV-2 infection after optimizing the COVID-19 response in China.

**Characteristic**		**Depression**, ***n*** **(%)**		
	**Total (*****n** =* **2,332), No. (%)**	**No (*****n** =* **1,508)**	**Yes (*****n** =* **824)**	***P*** **-value**
**Age**				0.016
≤ 24	993 (42.6)	672 (67.7)	321 (32.3)	
25–34	736 (31.6)	449 (61.0)	287 (33)	
≥35	603 (25.9)	387 (64.2)	216 (35.8)	
**Gender**				0.114
Male	842 (36.1)	562 (66.7)	280 (33.3)	
Female	1,490 (63.9)	946 (63.5)	544 (36.5)	
**Ethnicity**				0.456
Han	1,485 (63.7)	952 (64.1)	533 (35.9)	
Zhuang and other	847 (36.3)	556 (65.6)	291 (34.4)	
**Education**				0.034
High school or below	396 (34)	237 (59.8)	159 (40.2)	
College or undergraduate	1,445 (35)	936 (64.8)	509 (35.2)	
Postgraduate	491 (21.1)	335 (68.2)	156 (31.8)	
**Monthly income**				0.158
<2,000	976 (41.9)	652 (66.8)	324 (33.2)	
2,000–4,999	694 (29.8)	433 (62.4)	261 (37.6)	
≥5,000	662 (28.4)	423, (63.9)	239 (36.1)	
**Marital status**				0.664
Married	937 (40.2)	601 (64.1)	336 (35.9)	
Single	1,395 (59.8)	907 (65.0)	488 (35.0)	
**Occupation**				<0.001
Farmer or worker	196 (8.4)	89 (45.4)	107 (54.6)	
Company or government employee	716 (30.7)	485 (67.7)	231 (32.3)	
Business or self-employed	353 (15.1)	227 (64.3)	126 (35.7)	
Unemployed	185 (9.9)	103 (55.7)	82 (443)	
Student	882 (37.8)	604 (68.5)	278 (31.5)	
**Current residence area**				<0.001
Urban	1,855 (79.5)	1,231 (66.4)	624 (33.6)	
Rural	477 (20.5)	277 (58.1)	200 (41.9)	
**Residential status**				0.600
Live alone	408 (17.5)	255 (62.5)	153 (37.5)	
Live with family	1,512 (64.8)	984 (65.1)	528 (34.9)	
Live with others	412 (17.7)	269 (65.3)	143 (34.7)	
**The home-to-healthcare facility commute time**				<0.001
<30 mins	1,699 (72.9)	1,160 (68.3)	539 (31.7)	
≥ 30 mins	633 (27.1)	348 (55.0)	285 (45.0)	
**Chronic diseases**				<0.001
No	2,004 (85.9)	138 (42.1)	190 (57.9)	
Yes	328 (14.1)	1,370 (68.4)	634 (31.6)	
**Smoking**				<0.001
No	1,916 (82.2)	1,288 (67.2)	628 (32.8)	
Yes	416 (17.8)	220 (52.9)	196 (47.1)	
**Drinking**				0.001
No	1,744 (74.8)	1,160 (66.5)	584 (33.5)	
Yes	588 (25.2)	348 (59.2)	240 (40.8)	
**The interruption of physical exercise**				0.839
No	837 (35.9)	539 (64.4)	298 (35.6)	
Yes	1,495 (64.1)	969 (64.8)	526 (35.2)	
**Sleep difficulties**				<0.001
No	1,437 (61.6)	1,074 (74.7)	363 (25.3)	
Yes	895 (38.4)	434 (48.5)	461 (51.5)	
**Changes in medical expenses**				<0.001
The same as before	1,048 (44.9)	736 (70.2)	312 (29.8)	
More than before	1,197 (51.3)	734 (61.3)	463 (38.7)	
Less than before	87 (3.7)	38 (43.7)	49 (56.3)	
**Change in healthcare services**				<0.001
The same as before	1,221 (52.4)	856 (70.1)	365 (29.9)	
More than before	1,000 (42.9)	599 (59.9)	401 (40.1)	
Less than before	111 (4.8)	53 (47.7)	58 (52.3)	
**COVID-19 vaccination status**				0.017
Completed 0 doses	33 (1.4)	17 (51.1)	16 (48.5)	
Completed 1 dose	33 (1.4)	14 (42.4)	19 (57.6)	
Completed 2 doses	298 (12.8)	184 (61.7)	114 (38.3)	
Completed 3 doses	1,783 (76.5)	1,168 (65.5)	615 (34.5)	
Completed 4 doses	185 (7.9)	125 (67.6)	60 (32.4)	
**High consumption of COVID-19-related news**				0.791
No	522 (22.4)	1,173 (64.8)	637 (35.2)	
Yes	1,810 (77.6)	335 (64.2)	187 (35.8)	
**Perception of COVID-19**				0.006
Serious infectious disease	1,977 (84.8)	1,272 (64.3)	705 (35.7)	
Common infectious disease	289 (12.4)	203 (70.2)	86 (29.8)	
I don't know	66 (2.8)	33 (50.0)	33 (50.0)	
**Re-infection with SARS-CoV-2**				<0.001
No	2,133 (91.5)	82 (41.2)	117 (58.8)	
Yes	199 (8.5)	1,426 (66.9)	707 (33.1)	
**Length of COVID-19 symptoms**				0.003
≤ 6 days	903 (38.7)	592 (65.6)	311 (34.4)	
7–12 days	596 (25.6)	412 (69.1)	184 (30.9)	
≥13 days	833 (35.7)	504 (60.5)	329 (39.5)	
**Inpatient treatment for COVID-19**				<0.001
Yes	185 (7.9)	51 (27.6)	134 (72.4)	
No	2,147 (92.1)	1,457 (67.9)	690 (32.1)	

Table 3 presents the associated factors related to depression symptoms. Binary logistic regression analysis revealed that several factors were associated with an increased risk of depression symptoms. These factors included sleep difficulties (OR, 2.84; 95% CI, 2.34–3.44), chronic diseases (OR, 2.15; 95% CI, 1.64–2.82), inpatient treatment for COVID-19 (OR, 3.24; 95% CI, 2.19–4.77), COVID-19 symptoms more than 13 days (OR, 1.30; 95% CI, 1.04–1.63), re-infection with SARS-CoV-2 (OR, 1.52; 95% CI, 1.07–2.15), and the increase in demand for healthcare services (OR, 1.32; 95% CI, 1.08–1.61).

**Table 3:** Correlates of depression symptoms among participants with Self-reported SARS-CoV-2 infection after optimizing the COVID-19 response in China.

**Variables**	**cOR (95% CI)**	***P*-value**	**aOR (95% CI)**	***P*-value**
**Age**				
≤ 24	1 (ref)		1 (ref)	
25–34	1.34 (1.09–1.63)	0.004	1.09 (0.84–1.42)	0.495
≥35	1.17 (0.94–1.45)	0.152	0.67 (0.49–0.91)	0.012
**Occupation**				
Student	1 (ref)		1 (ref)	
Farmer or worker	2.61 (1.91–3.58)	<0.001	1.81 (1.21–2.72)	0.004
Company or government employee	1.04 (0.84–1.28)	0.751	1.01 (0.76–1.42)	0.942
Business or self-employed	1.24 (0.98–1.57)	0.072	1.04 (0.76–1.42)	0.827
Unemployed	2.96 (1.73–5.05)	<0.001	1.61 (1.08–2.40)	0.019
**Current residence area**				
Urban	1 (ref)		1 (ref)	
Rural	1.42 (1.16–1.75)	0.01	1.24 (0.98–1.58)	0.076
**The home-to-healthcare facility commute time**				
<30 mins	1 (ref)		1 (ref)	
≥ 30 mins	1.76 (1.46–2.12)	<0.001	1.23 (0.99–1.52)	0.058
**Chronic diseases**				
No	1 (ref)		1 (ref)	
Yes	2.98 (2.34–3.78)	<0.001	2.15 (1.64–2.82)	<0.001
**Changes in healthcare services**				
The same as before	1 (ref)		1 (ref)	
More than before	1.57 (1.32–1.87)	<0.001	1.32 (1.08–1.61)	0.005
Less than before	2.57 (1.73–3.79)	<0.001	1.54 (0.99–2.39)	0.053
**Sleep difficulties**				
No	1 (ref)		1 (ref)	
Yes	3.14 (2.63–3.75)	<0.001	2.84 (2.34–3.44)	<0.001
**Re-infection with SARS-CoV-2**				
No	1 (ref)		1 (ref)	
Yes	2.88 (2.14–3.87)	<0.001	1.52 (1.07–2.15)	0.018
**Length of COVID-19 symptoms**				
≤ 6 days	1 (ref)		1 (ref)	
7–12 days	0.85 (0.68–1.06)	0.151	0.83 (0.65–1.06)	0.131
≥13 days	1.24 (1.02–1.51)	0.029	1.30 (1.04–1.63)	0.022
**Inpatient treatment for COVID-19**				
No	1 (ref)		1 (ref)	
Yes	5.55 (3.97–7.76)	<0.001	3.24 (2.19–4.77)	<0.001

Figure 2 provides a summary of the self-reported COVID-19 symptoms among participants. The most common symptom was cough/runny nose (72.3%) among infected individuals (Figure 1A), followed by fever (71.4%), fatigue/drowsiness (65.7%), and headache/dizziness (58.6%). Regarding the reasons for inpatient treatment (Figure 1B), convulsions and high fever are the most common symptoms for inpatients, reported by more than 40% of those affected, followed by dyspnea (35.6%) and twitching (30.2%).

Figure 3A illustrates barriers to accessing healthcare services among participants with depressive symptoms. More than half of the participants (58.0%) reported experiencing a shortage of medication supply. Additionally, over one-third of participants reported longer waiting times for doctors (36.7%) and a shortage of medical staff (32.5%) compared to the past. Furthermore, 27.4% stated that they were required to provide a negative SARS-CoV-2 nucleic acid test result to access hospital services.

In addition, we investigated the reasons for delaying or canceling medical appointments for participants with depression symptoms. As shown in Figure 3B, 43.2% of the participants reported that they fear of causing SARS-CoV-2 infections to others after SARS-CoV-2 infection. Meanwhile, 44.3% expressed concerns that a hospital visit might increase the risk of re-infection with SARS-CoV-2. Approximately one-third of the participants reported financial difficulties in seeking medical care. A total 27.4% of the participants reported that the communication time with the doctor was insufficient to meet their needs.

## Discussion

We investigated the prevalence of depression symptoms and their correlates among individuals with SARS-CoV-2 infection after optimizing the COVID-19 response in China. We found that the prevalence of depression symptoms was 35.3%. Moreover, participants with sleep difficulties, chronic illnesses, inpatient treatment for COVID-19, symptoms duration for more than 13 days, re-infection with SARS-CoV-2 and the increased demand for healthcare services were associated with depressive symptoms. Additionally, the findings also revealed certain barriers that participants faced in accessing health care services. Thus, it is necessary for health professionals to implement targeted psychological interventions for high-risk populations.

Little evidence on how COVID-19 optimization policies influence depression symptoms among those infected in China. We found that the prevalence of depression symptoms was 35.3% which was similar to the study conducted in Pokhara (38) and China (25). The potential for temporary unemployment, reduced salary, and strained medical resources during the early stages of implementing the COVID-19 optimization policy may exacerbate psychological distress among the public. Besides, the physical distress caused by SARS-CoV-2 may bring significant emotional and psychological. That's why the COVID-19 pandemic continues to put a substantial burden on mental health. Nevertheless, it is noteworthy that certain studies have reported a higher prevalence of depression symptoms than the present study (39, 40). A study conducted in Shanghai showed that the prevalence of depression symptoms was 82% among infected individuals before the optimization of the COVID-19 response in China (28). The low prevalence of depression symptoms in this study may be attributed to the following reasons: First of all, increased awareness of COVID-19 illness (e.g., mitigated the risk of disease progression) and the adaptation to the pandemic, thereby might reduce the fear of COVID-19. Secondly, the optimal policies decreased the mobility restrictions (24), thereby effectively alleviating loneliness in the public. Moreover, those with asymptomatic or mild symptoms were permitted to self-isolate at home rather than being subjected to compulsory isolation (41), potentially easing their anxiety and worries. This low prevalence of depressive symptoms suggests the COVID-19 optimal policy in China has effectively reduced the negative impact of COVID-19 on mental health in the public. Nonetheless, mental health problems among SARS-CoV-2 infected individuals still warrant attention (24, 41).

Specifically, our study revealed that individuals aged 35 and above had a lower risk of developing depression symptoms during the pandemic compared with the 18–24 age group. Our findings align with prior research that has emphasized age-related disparities in the prevalence of depression symptoms (10, 18, 42). A study conducted at the Shahroud University of Medical Sciences in northeastern Iran revealed a substantial increase in depression scores among students after COVID-19 outbreak (43). In this study, a significant proportion of participants in the 18–24 age groups were students, who may face potential ongoing challenges, including prolonged distance learning, school closures, and exam delays. Therefore, it is advisable to pay attention to the mental health of individuals aged 18–24. Educational institutions should implement targeted mental health interventions to restore the mental health (44).

In line with previous studies, sleep difficulties were associated with depression symptoms during COVID-19 (10, 45). Almost all of the participants in this study reported experiencing COVID-19 symptoms (such as fever, cough, dyspnea, and fatigue). The severity of COVID-19 may cause difficulty falling asleep, subsequently increasing the risk of depressive symptoms. Furthermore, our study found a high proportion of participants experiencing sleep difficulties who expressed heightened concerns about the epidemic-related information and perceived COVID-19 as a severe disease (Supplementary Table 2). Thus, we hypothesized that their excessive apprehension negatively affected their sleep quality, potentially contributing to the persistence or worsening of depressive symptoms. Therefore, it is essential for individuals with sleep difficulties to seek mental health services or treatment to better cope with the challenges associated with sleep problems.

Furthermore, our findings are consistent with previous research suggesting an increased risk of depression symptoms with chronic disease (46, 47). Our study revealed that individuals with chronic diseases exhibited a high consumption of COVID-19-related news (Supplementary Table 2). Studies conducted in both China (48) and Japan (49) consistently indicated that patients with chronic diseases are at a higher risk of developing severe COVID-19, suggesting that the severity of the illness may worsen mental health. Additionally, previous reports consistently indicated that individuals with comorbidities such as diabetes (50), heart failure (51), chronic dialysis patients (52), HIV infection (53), chronic lung disease (54), chronic kidney diseases (55) and malignancies (56) experience significantly high mortality when infected with SARS-CoV-2. Therefore, an increased risk of depressive symptoms among SARS-CoV-2 infected individuals with chronic disease may be attributed to the fear of underlying health risks and the elevated mortality associated with COVID-19. To effectively address these challenges, it is crucial to develop personalized strategies and provide mental health services for patients with chronic disease when they are infected with SARS-CoV-2.

The disruptions in healthcare utilization during the pandemic had a substantial impact on the symptoms of depression among patients with chronic diseases (57, 58). We found that approximately half of the respondents with depression symptoms reported shortages of medications. Furthermore, a large number of infected individuals avoided seeking medical services from hospitals because they fear of causing SARS-CoV-2 infections to others. Further analysis revealed that these individuals experienced a decreased healthcare service utilization (Supplementary Table 3), indicating that they indeed encountered difficulties in seeking medical care. These findings underscore the challenges regarding to healthcare services among patients with chronic diseases. Therefore, it is necessary to provide timely and uninterrupted access to medical care for individuals with chronic diseases during the pandemic.

In this study, 7.9% of participants were hospitalized due to severe symptoms. Surprisingly, we found that inpatient treatment for COVID-19 increased the risk of depression symptoms. This is supported by previous studies that high incidences of depression symptoms were reported among patients hospitalized with COVID-19 (59–62). A possible explanation may be the fear of the high mortality of COVID-19 (63), which might significantly contribute to an increased risk of developing depressive symptoms. Furthermore, research indicated that individuals who have been hospitalized with COVID-19 are at a high risk of experiencing post-COVID-19 syndrome (64–67), potentially reflecting poor prognosis which may cause worry and stress among inpatients. Therefore, timely communication with patients about their health status and progress of treatment is necessary to alleviate their fear of uncertainty. Also, health professionals should provide effective psychological support and treatment to reduce mental burdens in patients hospitalized with COVID-19.

This study has several limitations. Firstly, we could not confirm the causal relationship due to the limitation of a cross-sectional design. Future studies with longitudinal designs would be necessary. Secondly, the self-reported data might introduce recall bias, which may affect the reliability of the findings. Thirdly, it is important to acknowledge that the findings may be limited in generalizability due to the small sample size. Moreover, it is crucial to acknowledge that we did not collect data to assess other psychological factors such as anxiety, trauma, and stress. This is an important limitation of this study, as these mental health issues may also play significant roles in mental health among participants. Lastly, the use of PHQ-9 scales may overestimate the prevalence of depressive symptoms (68), as the clinical diagnosis is typically required for accurate determination.

## Conclusion

This study revealed a moderate prevalence of depressive symptoms among individuals infected with SARS-CoV-2 after optimizing the COVID-19 response in China. It is necessary to provide mental health services and psychological interventions for the at-risk groups, including individuals with sleep difficulties, chronic diseases, inpatient treatment for COVID-19, long COVID-19 symptoms duration, and re-infection with SARS-CoV-2. Furthermore, health policymakers should formulate policies and interventions for responding to the mental health challenges for the future pandemics similar to SARS-CoV-2.

## Data availability statement

The raw data supporting the conclusions of this article will be made available by the authors, without undue reservation.

## Ethics statement

The study was approved by the Medical Ethics Committee of Guangxi Medical University (20220206). All participants were provided with online informed consent. The studies were conducted in accordance with the local legislation and institutional requirements.

## Author contributions

LW: Writing—original draft. JQ: Writing—original draft. ZLin: Investigation, Visualization, Writing— original draft. XH: Investigation, Supervision, Writing—review & editing. JH: Investigation, Methodology, Writing—review & editing. DY: Project administration, Writing—review & editing. FZ: Software, Writing— review & editing. SL: Supervision, Writing—review & editing. PC: Writing—review & editing. ML: Project administration, Writing—review & editing. TL: Data curation, Writing—review & editing. RZ: Writing—review & editing. SZ: Writing—review & editing. CQ: Writing—review & editing. ZLi: Writing—review & editing. YY: Investigation, Writing—review & editing. HP: Writing—review & editing. MZ: Writing—review & editing. XW: Writing—review & editing. JJ: Supervision, Writing—review & editing. HL: Funding acquisition, Writing—review & editing. LY: Funding acquisition, Supervision, Writing— review & editing. BL: Funding acquisition, Supervision, Writing—review & editing.
