# Supplementary material for: Prevalence of depressive symptoms and correlates among individuals who self-reported SARS-CoV-2 infection after optimizing the COVID-19 response in China
Source: Front Public Health. 2024 Jan 8;11:1268799. doi: 10.3389/fpubh.2023.1268799 (PMC10800514; doi:10.3389/fpubh.2023.1268799)
Supplement: Supplementary file 1 [file Table_1.DOCX]

Supplementary Material

# Supplementary Figures and Tables

**Table S1** Correlates of depression symptoms among participants after optimizing the COVID-19 response in China

| **Variables** | **cOR (95% CI)** | ***P*** value | **aOR (95% CI)** | ***P*** value |
| --- | --- | --- | --- | --- |
| **Age** |  |  |  |  |
| ≤24 | 1 (ref) |  | 1 (ref) |  |
| 25-34 | 1.28(1.06-1.54) | 0.010 | 1.13(0.89-1.44) | 0.305 |
| ≥35 | 1.11(0.91-1.36) | 0.295 | 0.70(0.53-0.0.94) | 0.016 |
| **Occupation** |  |  |  |  |
| Student | 1 (ref) |  | 1 (ref) |  |
| Farmer or worker | 2.36(1.77-3.16) | ＜0.001 | 1.74(1.21-2.5) | 0.003 |
| Company or government employee | 0.99(0.82-1.22) | 0.995 | 0.90(0.69-1.18) | 0.443 |
| Business or self-employed | 1.25(1.00-1.56) | 0.046 | 1.03(0.78-1.35) | 0.844 |
| Unemployed | 2.26(1.59-4.11) | ＜0.001 | 2.11(1.22-3.65) | 0.008 |
| **Smoking** |  |  |  |  |
| No | 1 (ref) |  | 1 (ref) |  |
| Yes | 1.70(1.39-2.07) | ＜0.001 | 1.55(1.23-1.95) | ＜0.001 |
| **Chronic diseases** |  |  |  |  |
| No | 1 (ref) |  | 1 (ref) |  |
| Yes | 3.02(2.42-3.78) | ＜0.001 | 2.62(2.04-3.36) | ＜0.001 |
| **Changes in medical expenses** |  |  |  |  |
| The same as before | 1 (ref) |  | 1 (ref) |  |
| More than before | 1.64(1.36-1.89) | ＜0.001 | 1.36(1.13-1.64) | 0.001 |
| Less than before | 2.96(1.99-4.40) | ＜0.001 | 1.83(1.18-2.86) | 0.008 |
| **Sleep difficulties** |  |  |  |  |
| No | 1 (ref) |  | 1 (ref) |  |
| Yes | 3.45(2.92-4.08) | ＜0.001 | 3.17(2.65-3.79) | ＜0.001 |
| **The home-to-healthcare facility commute time** | | | |  |
| ＜ 30 mins | 1 (ref) |  | 1 (ref) |  |
| ≥ 30 mins | 1.73(1.45-2.05) | ＜0.001 | 1.35(1.11-1.64) | 0.003 |
| **Attitudes towards COVID-19** | | | |  |
| Serious infectious disease | 1 (ref) |  |  |  |
| Common infectious disease | 0.77(0.59-0.99) | 0.045 | 0.92(0.69-1.21) | 0.534 |
| I don't know | 1.49(0.97-2.28) | 0.070 | 1.66(1.02-2.71) | 0.041 |
| **Self-reported COVID-19 infection** | | | |  |
| No | 1 (ref) |  | 1 (ref) | ＜0.001 |
| Yes | 2.29(1.76-2.98) | ＜0.001 | 2.03(1.53-2.70) |  |

**Table S2** Difference in distribution of sleep status on high consumption of COVID-19-related news and perception of COVID-19

| **Characteristic** | **Total (n = 2332), No.(%)** | **Sleep difficulties, No.(%)** | | ***P* value** |
| --- | --- | --- | --- | --- |
|  |  | **No(n = 1437)** | **Yes (n = 895)** |  |
| **High consumption of COVID-19-related news** | | |  | ＜0.001 |
| Yes | 1810(77.6) | 1070(74.5) | 740(82.7) |  |
| No | 522(22.4) | 367(25.5) | 155(17.3) |  |
| Perception of COVID-19 | |  |  | ＜0.001 |
| Very serious infectious disease | 1977(84.8) | 1185(82.5) | 792(88.5) |  |
| Common infectious disease | 289(12.4) | 208(14.5) | 81(9.1) |  |
| I don't know | 66(2.8) | 44(3.1) | 22(2.5) |  |

**Table S3** Difference in distribution of sleep status on healthcare-related changes

| **Characteristic** | **Total (n = 2332), No.(%)** | **Chronic diseases, No.(%)** | | ***P value*** |
| --- | --- | --- | --- | --- |
|  |  | **No(n = 2004)** | **Yes(n = 328)** |  |
| Changes in medical expenses | | |  | ＜0.001 |
| The same as before | 1048(44.9) | 916(45.7) | 132(40.2) |  |
| More than before | 1197(51.3) | 1029(51.3) | 168(51.2) |  |
| Less than before | 87(3.7) | 59(2.9) | 28(8.5) |  |
| Change in healthcare services | | |  | 0.039 |
| The same as before | 1221(52.4) | 1060(52.9) | 161(49.1) |  |
| More than before | 1000(42.9) | 870(43.4) | 130(39.6) |  |
| Less than before | 111(4.8) | 74(3.7) | 37(11.3) |  |
